# Lysozyme Functionalized Alginate-Chitosan Beads and Films for Different Release Applications

**DOI:** 10.3390/gels12010066

**Published:** 2026-01-11

**Authors:** Beatriz Moutinho, Natalia Pyra, Zuzanna Styrna, Maria Emilia Rosa, Maria H. L. Ribeiro

**Affiliations:** 1 Research Institute of Medicines, Faculty of Pharmacy, Universidade de Lisboa, Av. Prof. Gama Pinto, 1649-003 Lisboa, Portugal; beatriz.moutinho@edu.ulisboa.pt (B.M.); nataliapyra10@gmail.com (N.P.); sstyrnazuzanna@gmail.com (Z.S.); 2 Faculty of Chemical Technology, Poznan University of Technology, 5 M. Skłodowska-Curie Square, 60-965 Poznan, Poland; 3 Instituto de Engenharia Mecânica (IDMEC), Instituto Superior Técnico, Universidade de Lisboa, Av. Rovisco Pais, 1049-001 Lisboa, Portugal; emilia.rosa@ist.utl.pt

**Keywords:** lysozyme, hydrogels, alginate, chitosan, anti-inflammatory activity, antioxidant activity

## Abstract

The main goal of this work was to develop nanoparticles of lysozyme (Lys) for biological and biomedical applications. The developed biosystems were based on Lys-loaded calcium alginate 2% and chitosan 1% beads and films with different concentrations of each polymer. Encapsulation efficiency was 100%. The ratio of adsorbed Lys on the films, Lys activity, and the release profile of Lys were measured using water and buffer solution at pH similar to the environment of cancer cells, at a controlled temperature of 37 °C and a constant speed, to assess the efficacy of the encapsulation process. Lys antimicrobial activity was assessed using *Micrococcus lysodeikticus*. Moreover, the anti-inflammatory and antioxidant properties of the developed biosystems were also evaluated. The anti-inflammatory activity of Lys released from calcium alginate 2%-chitosan 1% beads loaded with Lys was about 99%. These findings highlight the potential of the developed beads and films for biomedical applications, particularly in antimicrobial and anti-inflammatory therapies.

## 1. Introduction

Alginate (AL) and Chitosan (CS) are well-known natural polymers extensively studied for their properties, making them ideal candidates for delivery systems. Alginate, an abundant natural polymer, is an anionic hydrophilic heteropolysaccharide found in the capsular polysaccharides of certain soil bacteria and as a structural component of brown seaweed (Phaeophyceae) [1]. Sodium alginate, one of the most widely used alginates, is particularly valued for its flexibility, biocompatibility, biodegradability, low cost, and natural abundance. These properties, combined with its ability to form gels in the presence of polyvalent cations, make it highly suitable for producing particles and nanoparticles for various applications. Alginate’s applications are widespread, particularly in drug delivery systems at the nanoscale. It minimizes side effects, encapsulates or releases drugs and cells, and serves as a component in packaging films or edible fibers [2,3]. For example, calcium alginate nonwoven dressings, which exchange ions with wound fluid, are widely used for infected or exuding surgical wounds. These dressings form a highly absorbent, soluble gel that maintains a moist physiological environment, promoting new epidermal growth [4] and accelerating healing [5]. Additionally, alginate is employed as a biomaterial in tissue reconstruction, including applications in teeth, bone, and cartilage, due to its mechanical stability and viscoelastic properties [6].

The process of alginate gel formation is complex and depends on various factors, including the type of alginate used, the degree of conversion to calcium alginate, the source of calcium ions (e.g., calcium chloride, phosphate, lactate, or acetate), and the preparation methods [7]. Studies using gel chromatography and electron microscopy have demonstrated that the pore sizes in alginate gels range from 5 to 200 nm.

Chitosan (CS) is a natural polysaccharide derived from chitin through chemical processes, primarily demineralization and deproteination. Chitin can be obtained from natural sources such as shrimp and crab shells or fungi, as well as from purified synthetic substances. Chitosan’s biological attributes—antibacterial, anti-inflammatory, and antioxidant properties, along with its biodegradability and biocompatibility—make it widely applicable in the food, pharmaceutical, and biotechnology industries.

Several methods exist for producing chitosan nanoparticles, with ionic gelation emerging as a particularly promising technique. This method employs crosslinkers to create a permanent covalent network, enhancing the polymer’s mechanical properties, such as film strength, while allowing for free diffusion of water and bioactive substances [8]. Common crosslinkers include alginate, sodium tripolyphosphate (TPP), cinnamaldehyde, and glutaraldehyde [9].

Chitosan has demonstrated several advantageous properties for drug delivery applications. It protects molecules against the acidic environment of the stomach and enhances their absorption in the gastrointestinal tract [10]. An example of its application is the use of chitosan/alginate-based multilayers in controlled drug release. For instance, these multilayered systems have been shown to regulate the release of diclofenac from ophthalmic lenses, maintaining drug release for at least one week [11].

Lysozyme is an abundant basic protein found in various organs, tissues, and body fluids like tears, saliva, milk, serum, and mucus, functioning as an antimicrobial enzyme [12,13]. It catalyzes the hydrolysis of β(1,4) linkages between N-acetylmuramic acid and N-acetyl-D-glucosamine residues in peptidoglycan, a key structural component of the bacterial cell wall [14,15].

Lysozymes are widely spread, not only found in the animal kingdom but also in plants and bacteria [16]. However, they show diversity in characteristics like structure, catalytic mechanism, and immune function. Hence, lysozymes have been classified into three main families: chicken type (c-type), goose type (g-type), and invertebrate type (i-type). Other known types of lysozymes include the phage type, bacterial type, and plant type. The c-type lysozymes, with a molecular weight of approximately 11–15 kDa, are significantly smaller than the g-type lysozymes, which typically range from 20 to 22 kDa. Chicken (cLys) and human (hLys) lysozymes belong to the c-type lysozyme family as their main representatives. The cLys consists of 129 amino acid residues (14.3 kDa), while hLys consists of 130 amino acid residues (14.7 kDa) [12,16].

Lysozyme can be obtained from bird eggs, with chicken eggs being the most abundant [14,16]. Egg white is composed of 11% protein, with lysozyme accounting for 3.5% of the total protein content [12,17].

Recently, the hen egg-white lysozyme (HEWL) became the subject of interest among researchers due to its functional features, high accessibility, and relatively low cost [14]. Hen egg white lysozyme exhibits various biological activities, including antibacterial [18], antioxidant, antiviral [19] and anti-inflammatory properties. However, its antioxidant and anti-inflammatory potential has not yet been well studied [20]. The bacteriostatic and bactericidal properties of lysozyme have been applied in food preservation, as well as in pharmaceutical applications, human medicine, and veterinary medicine [16]. The lysozyme has shown antimicrobial potential, especially against Gram-positive bacteria, due to the hydrolyzation of the β-(1,4)-glycosidic linkages between N-acetylmuramic acid and N-acetylglucosamine of peptidoglycan chains. Peptidoglycan is a constituent of bacterial cell wall and can be disrupted by lysozyme leading to bacterial cell lysis or ultimately cell death [18].

Besides its antibacterial enzymatic activity, lysozyme also exhibits anti-inflammatory properties [20,21]. Hen egg white lysozyme has been shown to stimulate lymphocytes to produce antibodies. Furthermore, heat-treated lysozyme has been indicated to increase both antibacterial and immunostimulatory activities, with heat treatment during processing considered a key factor in boosting its immunostimulatory effects [22,23]. Lysozyme also exhibits antioxidant properties, attributed to certain regions of its amino acid sequence. The enzyme consists of an 18-amino acid domain that binds to glycation end products, which can produce highly reactive oxygen species, significantly influencing metabolism and the aging process in living organisms [14].

Previous studies explored the effect of different boronic acids for cross-linking PVA-lysozyme nanofilms and compared their effects on lysozyme cell viability [24]. However, the study of other polymers in lysozyme encapsulation and their antioxidant and anti-inflammatory activities remains to be addressed. This work aims to fill this gap, so, the main goal was to investigate the encapsulation of lysozyme (Lys) within beads and films made using individual polymers, as well as different blends of them, alginate and chitosan. This study was motivated by the critical biological significance of Lys and the well-established functionality of alginate and chitosan in drug delivery systems.

Therefore, the specific aims of this work included (i) characterization of AL-Lys and AL-CS-Lys beads and films; (ii) the evaluation of the encapsulation efficiency of Lys within beads and films; (iii) assessment of the release capacity of Lys from the delivery systems under controlled conditions; (iv) evaluation of the biological activity of Lys from beads and films of alginate, chitosan, or their blends.

## 2. Results and Discussion

### 2.1. Morphological Characterization of AL-Lys and AL-CS-Lys Beads and Films by SEM

The morphology of the calcium alginate-based systems (AL-Lys) and alginate-chitosan (AL-CS-Lys) containing lysozyme was investigated by scanning electron microscopy (SEM).

#### 2.1.1. Ca-Alginate Beads with Lysozyme

**Figure 1:**
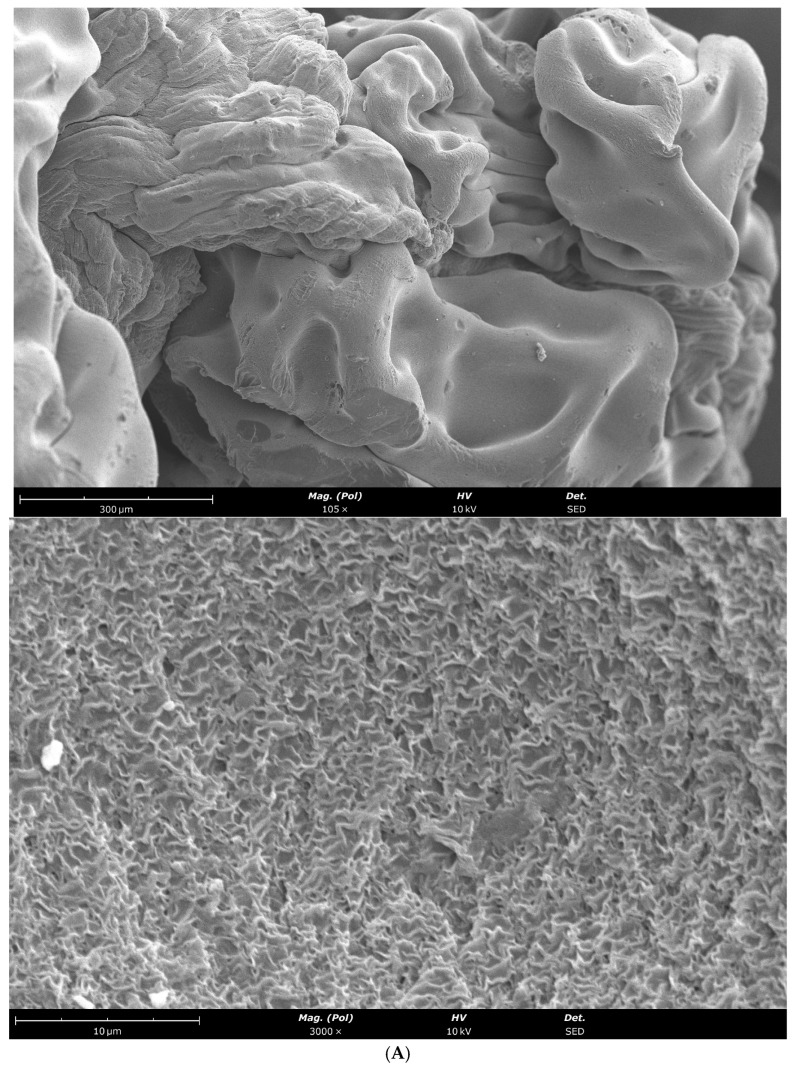

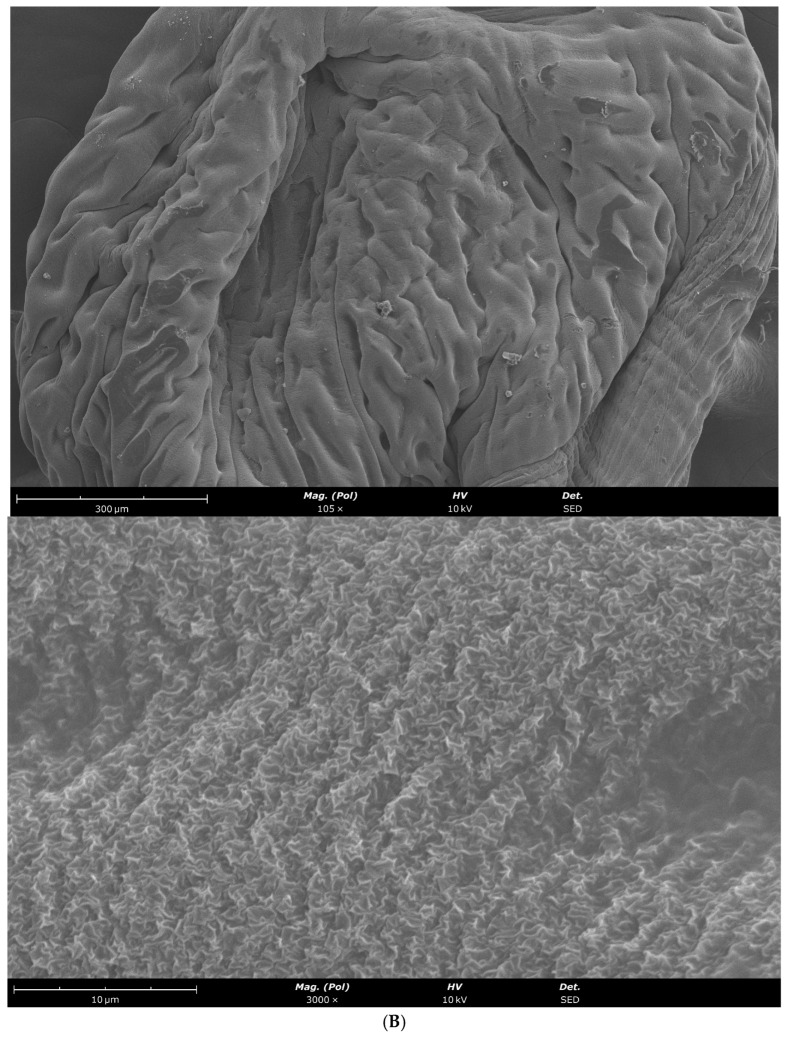

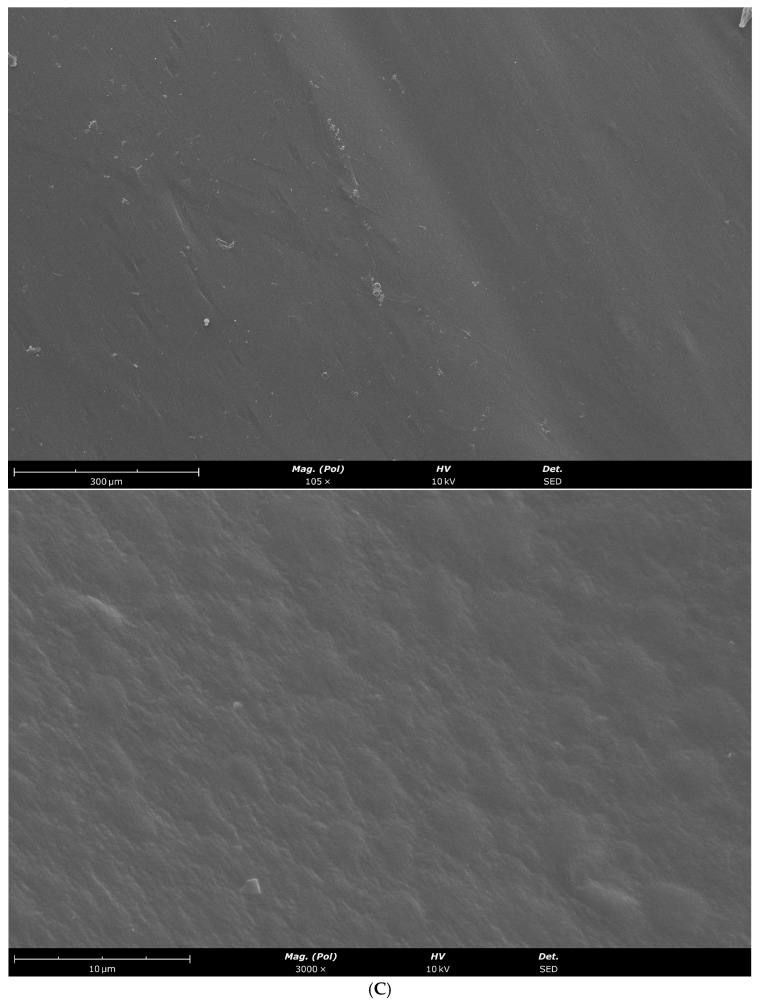

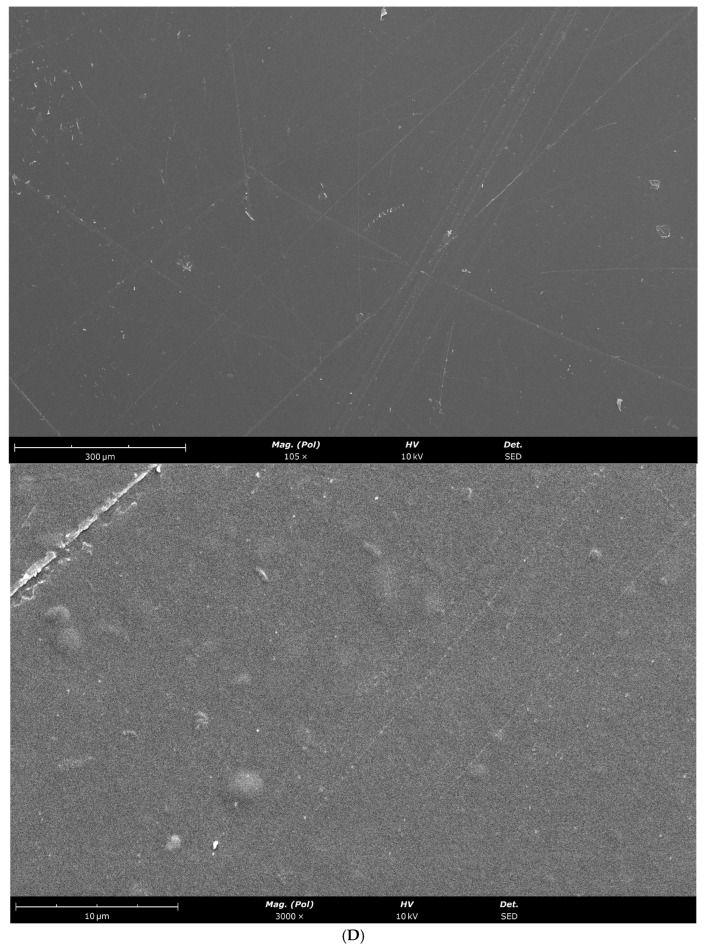
SEM images with magnification of 105× *g* and 3000× *g*: Beads of calcium-alginate 2% (**A**) and calcium-alginate 2%-chitosan 1% (**B**); Films of calcium-alginate 2% (**C**) and calcium-alginate 2%-chitosan 1% (**D**).

SEM micrographs (Figure 1A) show spherical Ca-alginate beads with an average diameter of approximately 500 µm. The bead surface appears rough and heterogeneous. At higher magnification, the internal structure reveals a granular morphology, with granules presenting characteristic dimensions on the order of 1 µm.

#### 2.1.2. Ca-Alginate/Chitosan Beads with Lysozyme

The Ca-alginate/chitosan beads containing lysozyme exhibit a reduced average diameter of approximately 300 µm (Figure 1B). The overall morphology is similar to that observed for Ca-alginate beads; however, the structure appears finer and more compact. The size of the small granules is estimated to be around 0.5 µm. The presence of chitosan results in a smoother and more uniform surface compared to beads composed solely of Ca-alginate.

#### 2.1.3. Ca-Alginate Films and Ca-Alginate/Chitosan Films with Lysozyme

Based on the SEM image (3000× *g* magnification, 10 µm scale bar) (Figure 1C,D), a continuous surface without marked heterogeneities is observed, displaying a rough texture with very subtle wavy/lamellar patterns and an absence of individual structures such as fibers, microspheres, nanotubes, or crystalline particles. Although the films appear more defined with Ca-alginate-chitosan (Figure 1D) compared to those observed in the absence of chitosan (Figure 1C). The observed surfaces are typical of dried polymer films, namely of alginate and chitosan.

#### 2.1.4. Comparison Between Beads and Films Morphologies

SEM analysis allowed a qualitative and semi-quantitative comparison between beads and films-based systems containing lysozyme. Bead formulations exhibited spherical morphologies, with a reduction in bead size and a smoother surface observed upon chitosan incorporation, suggesting enhanced polymer–polymer interactions and a denser network formation, which is consistent with the finer granular structure (granule size ≈ 0.5 µm) compared to Ca-alginate alone (granule size ≈ 1 µm).

In contrast, samples prepared under film-forming conditions did not display continuous or elongated fibrous structures, indicating that, under the experimental conditions employed, the system favors film formation rather than fiber assembly. The presence of chitosan in the film formulations led to the appearance of more defined and compact structures, suggesting a degree of structural stabilization. These observations highlight the strong influence of formulation composition and processing conditions on the resulting morphology. In conclusion, the images (Figure 1C,D) indicate the presence of a continuous polymeric film rather than a matrix of nanofibers or individualized particles.

### 2.2. Encapsulation Efficiency

Beads and films of calcium alginate 2%, loaded with lysozyme (Lys), were prepared following the procedures outlined in Section 4. To evaluate the encapsulation efficiency of Lys, it the enzyme was quantified in the solution of CaCl_2_ 2%, which was achieved using the Bradford method assay, a well-established method for protein quantification. The results indicated that no detectable enzyme was released into the CaCl_2_ 2% solution, indicating an encapsulation efficiency of approximately 100%. These outcomes established that the enzyme was entirely retained within the beads and the films of calcium alginate 2% and calcium alginate 2%-chitosan 1%, showing the efficacy of these encapsulation processes.

### 2.3. Lysozyme Activity Free and Encapsulated

**Figure 2:**
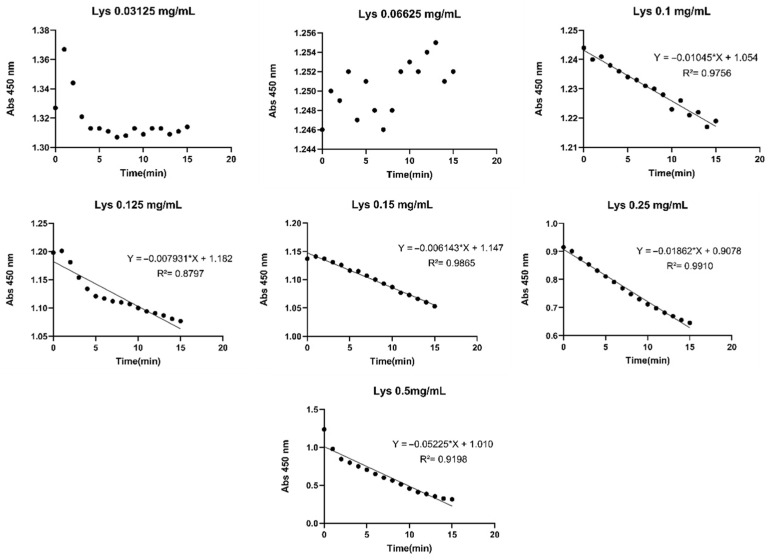
Lysozyme activity (lines) (0–15 min) (SD ± 0.005) using *Micrococcus lysodeikticus* (1 mg/mL) at different lysozyme concentrations evaluated at 450 nm. Black dots represent experimental data points obtained from triplicate measurements.

The enzymatic activity of lysozyme was based on the evaluation of the effect of its concentration on *Micrococcus lysodeikticus*. This approach enabled the quantification of the gradual reduction in optical density, at 450 nm, resulting from lysozyme-mediated degradation of peptidoglycan in *Micrococcus lysodeikticus*. Furthermore, it allowed for the establishment of a correlation between the degradation rate and lysozyme concentration. The results are presented in Figure 2, which depicts the absorbance values at 450 nm as a function of time.

At low lysozyme concentrations (0.03125 and 0.06625 mg/mL), no clear relationship between absorption and time (Figure 2) was observed to effectively assess the progression of enzymatic degradation and its associated antimicrobial activity. Lysozyme activity tended to decrease for high lysozyme concentrations. A possible explanation for this decrease was a self-inhibition mechanism.

**Figure 3:**
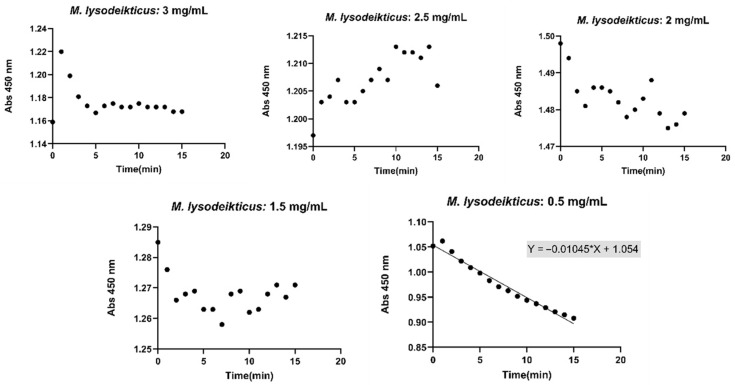
Lysozyme activity (Lines) (0–15 min) (SD ± 0.005), at 450 nm, using different concentrations of *Micrococcus lysodeikticus* and at a lysozyme concentration of 0.125 mg/mL. Black dots represent experimental data points obtained from triplicate measurements.

Different concentrations of *Micrococcus lysodeikticus* (3 mg/mL, 2.5 mg/mL, 2 mg/mL, 1.5 mg/mL, and 0.5 mg/mL) were analyzed, and variations in the slope were observed for the same lysozyme concentration. For instance, at a lysozyme concentration of 0.125 mg/mL, the corresponding slopes are shown in Figure 3. Based on these results, a *Micrococcus lysodeikticus* concentration of 0.5 mg/mL was selected for subsequent assays, as it provided the most consistent linearity across all lysozyme concentrations above 0.1 mg/mL.

**Figure 4:**
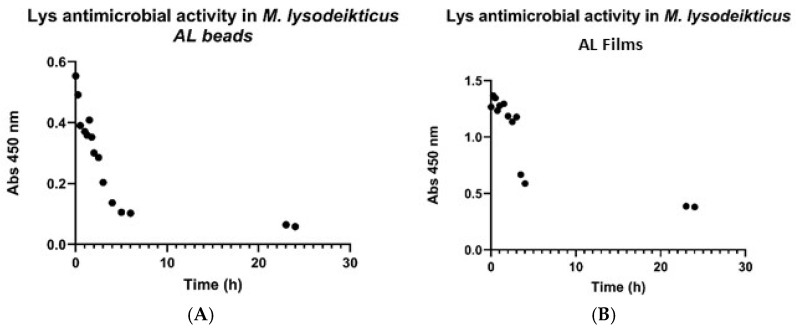
Antimicrobial activity of lysozyme in calcium alginate (2%): beads (**A**) and films (**B**). (SD ± 0.005).

The antimicrobial activity of lysozyme was subsequently evaluated in calcium alginate 2% beads and films, as shown in Figure 4. According to the results of Figure 4, lysozyme shows antimicrobial activity during the first five hours of the assay. During this period, a rapid and pronounced decrease in absorbance was observed, from 0.554 to 0.104, indicating that lysozyme effectively promotes the degradation of *Micrococcus lysodeikticus*. After 1.5 h, a logarithmic trend was detected. For the remainder of the assay, the *Micrococcus lysodeikticus* concentration stayed constant.

Similar effects were seen on the lysis of *Micrococcus lysodeikticus* for PVA-Lys nanofilms in the work of Guerra et al. 2023 [24] where the properties of hydrogel polyvinyl alcohol and chitosan electrospun nanofilms were studied. In the first two hours, there was a significant decrease in absorbance, and twenty percent of the *Micrococcus lysodeikticus* degradation continued to occur 24 h later. Also, the results of the work of Wu et al. 2017 [25] suggested that the integration of lysozyme into chitosan nanoparticles enhanced the antibacterial activity against *E. coli* and *B. subtilis*, which may show great potential for use in the food industry and other applications in the form of direct addition or incorporation into packaging [25].

### 2.4. Lysozyme Release Assays from Beads and Films of Calcium Alginate 2%

**Figure 5:**
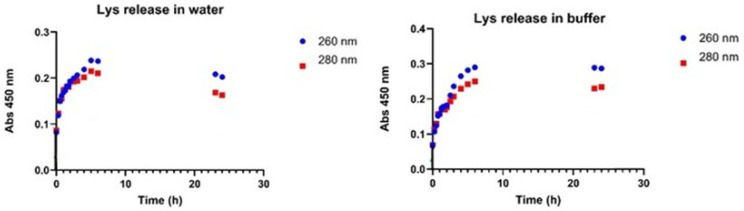
Release of Lys from calcium alginate 2% beads in water, and buffer pH 7.4 at 37 °C and 150 rpm (SD ± 0.005).

Release assays using Lys encapsulated in calcium alginate beads were performed using water (pH = 7.0) and buffer (pH = 7.4) for 24 h and measured at 260 nm and 280 nm at different times. In Figure 5, the results indicated that pH did not influence the initial release of Lys. The similarity in the slopes of the release profiles during the first six hours suggests no significant difference between the buffer and water environments. However, the amount of Lys released within the first six hours varied, namely the concentration in water was approximately 0.19 mg/mL, while in the buffer it was slightly higher, 0.23 mg/mL. After 23–24 h, the concentration of Lys in the water solution decreased over time, whereas the concentration in the buffer solution remained stable throughout the 24 h experiment. This performance suggested that the buffer maintained the enzyme’s stability more effectively than water over the experimental period. Under physiological conditions, the detection of particles was performed by evaluation of sedimentation over time.

**Figure 6:**
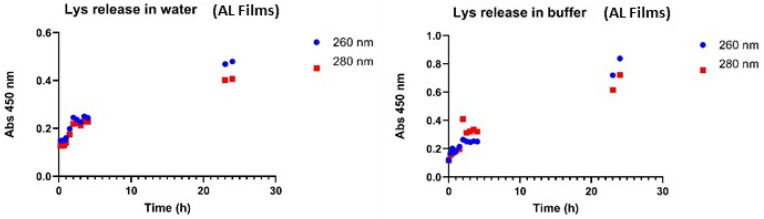
Lys release from calcium alginate 2% films in water and buffer (pH 7.4) (SD ± 0.005).

Lys release in water and buffer (pH 7.4) in films was evaluated (Figure 6). After 24 h the Lys release from the films in buffer was almost two-fold than in water (Figure 6).

### 2.5. Lysozyme Release Assays from Beads and Films of Calcium Alginate 2%-Chitosan 1%

**Figure 7:**
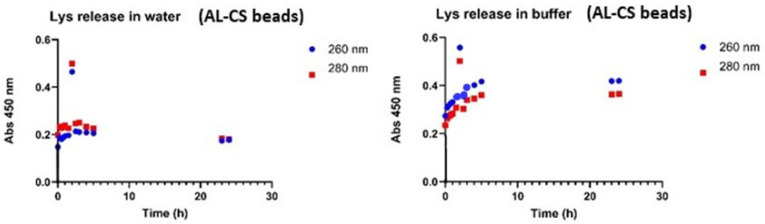
Release of Lys from beads of calcium alginate 2%-chitosan 1% in water and buffer 7.4, at 37 °C and 150 rpm (SD ± 0.005).

**Figure 8:**
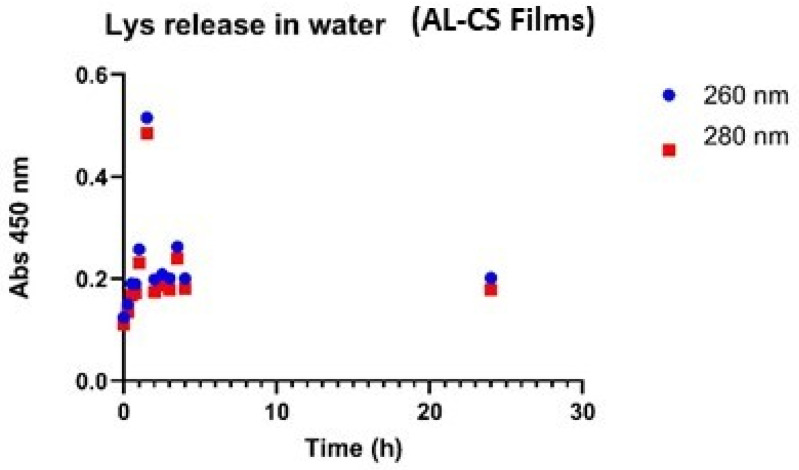
Lys release from Calcium Alginate 2%-Chitosan 1% films in water (SD ± 0.005).

Lys was released from beads of calcium alginate 2%-chitosan 1% in water (pH = 7.0) and buffer (pH = 7.4) for 24 h. The Lys in solution was measured at 260 and 280 nm (Figure 7). The results indicated that the amount of Lys released in water and buffer differs significantly (Figure 7) and that the pH difference also affects the release rate. The maximum concentration release of Lys in water was 0.17 mg/mL, and in the buffer was 0.32 mg/mL. Beads with alginate and chitosan showed a better profile of release of Lys when compared to alginate beads, 0.23 mg/mL vs. 0.32 mg/mL. The release of Lys in water from films was evaluated (Figure 8).

The data indicate that the pH difference between the buffer and water had no impact on the initial release of Lys. After the first five hours, the Lys concentration in water was approximately 0.18 mg/mL. At the 23- and 24 h time points, the Lys concentration in the buffer was around 0.7 mg/mL, whereas in water, it ranged between 0.3 and 0.55 mg/mL, indicating more efficient release in the buffer environment.

Beads of calcium alginate 2% and chitosan 1% reached a maximum Lys concentration of 0.23 mg/mL, while beads containing only calcium alginate 2% achieved 0.32 mg/mL. Both values were significantly lower than those observed in films. The release profile of Lys from calcium alginate-chitosan films in water was comparable to that of calcium alginate films previously reported.

Notably, both solutions displayed a significant absorbance after two hours. This phenomenon has been previously observed in beads composed of this polymer combination. The Bradford assay was performed to analyze the protein in the reaction medium, providing insight into whether film degradation occurred during the test and contributed to the observed burst release at the 2 h mark.

According to the study of Guerra et al., 2023 [24], PVA/CS films with compositions of 90:10 and 70:30 exhibited similar release profiles, suggesting that the polymer composition had minimal impact on Lys release. For both PVA and PVA/CS nanofibers, a noticeable enzyme release was observed after 30 min, which persisted for 48 h in PVA fibers and 24 h in PVA/CS fibers.

### 2.6. Anti-Inflammatory Activity

**Figure 9:**
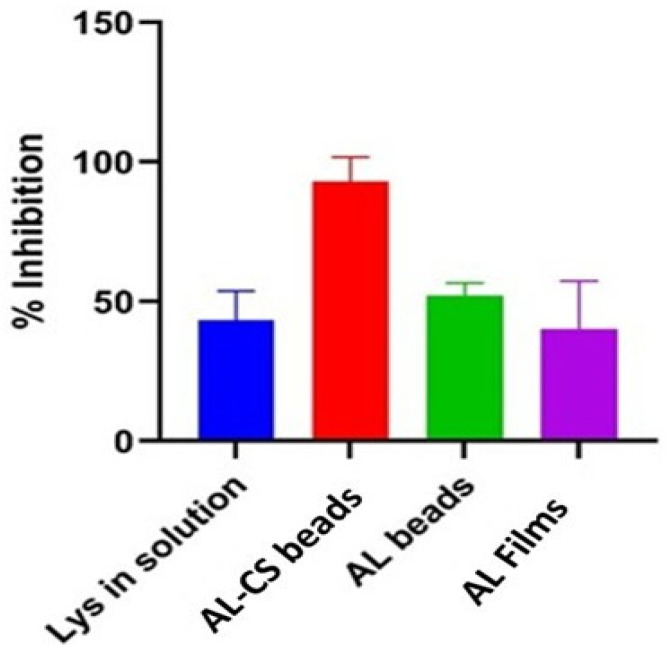
Anti-inflammatory activity of lysozyme (Lys) in solution and released from beads and films of calcium alginate 2% (AL), calcium alginate 2% and chitosan 1% (AL-CS).

Due to the prominent antibacterial properties of hen egg white lysozyme, research about this enzyme has primarily focused on its antibacterial activities [26]. Limited attention has been given to exploring its potential in other biological activities, such as anti-inflammatory and antioxidant properties. In this work, additionally it was explored the anti-inflammatory and antioxidant activities of Lys in solution and encapsulated in calcium alginate 2% beads, films, and calcium alginate 2%-chitosan 1% beads. The results are displayed in Figure 9.

Lys solution with a concentration of 0.5 mg/mL, showed that the percentage of inhibition for protein denaturation was the higher, 55% (Figure 9). These values are in line with the potential of anti-inflammation shown for this enzyme in solution [27].

When Lys was encapsulated, the highest inhibition of protein denaturation (99%) was observed when a combination of 2% calcium alginate and 2% chitosan was used. This finding suggests that not only was the intrinsic anti-inflammatory activity of Lys preserved, but it was also enhanced by this delivery system. Additionally, these polymers possess inherent anti-inflammatory properties, which, in combination with Lys, provide a synergistic advantage in this formulation for controlled delivery.

Several studies demonstrated the anti-inflammatory activity of Lys in solution [28,29,30,31]. This activity is linked to gene regulation involving the proteins of the inflammatory pathway, such as tumor necrosis factor alpha (TNF-α) and interleukin 1 beta (IL-1β) [23]. In the work of [27] was discovered that Lys dose-dependently inhibits the classical pathway of serum complement activity, especially in pathologic settings. Complement systems continue to play a significant role in mucosal surface antimicrobial activity and modulate the anti-inflammatory response [27]. In another study [32], the Hen Egg Lysozyme (HEL) was evaluated as a potentially effective treatment option for Inflammatory Bowel Disease (IBD) in a porcine model of dextran sodium sulfate-induced colitis. The function of the epithelial barrier was restored, and DSS-induced inflammation was lessened by HEL. Moreover, HEL supplementation increased the expression of regulatory and anti-inflammatory genes while decreasing the expression of pro-inflammatory cytokines [32].

### 2.7. Antioxidant Activity 

The antioxidant activity of lysozyme encapsulated (0.5 mg/mL), was evaluated by DPPH method in the beads and the films of calcium alginate 2% and calcium alginate 2%-chitosan 1%, respectively.

The ability to neutralize the oxidant radical is evident; however, the reaction proceeds at a slow rate. The neutralization of the DPPH free radical may require an extended duration, potentially spanning several hours, as indicated by the gradual colour transition of the solution from purple to yellow. This slow-release behaviour could be harnessed for a lysozyme-controlled release delivery system.

**Figure 10:**
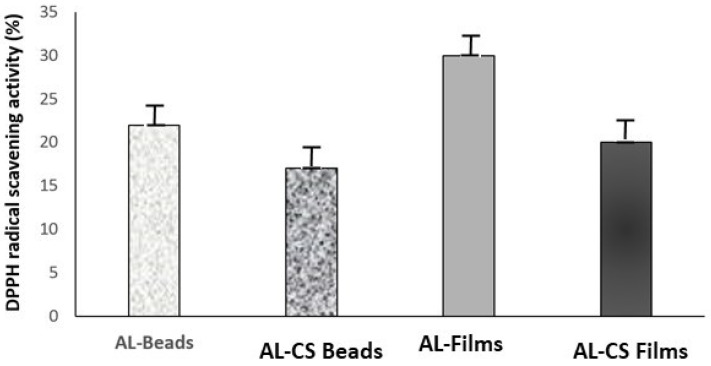
Antioxidant activity of Lys in calcium alginate 2% (AL-Beads), calcium alginate 2%-chitosan 1% (AL-CS beads), calcium alginate 2% (AL-Films), and calcium alginate 2%-chitosan 1% (AL-CS Films).

The lysozyme in calcium alginate 2% beads displayed a significant increase in the antioxidant activity when compared to the lysozyme in solution, 22% reduction in DPPH (Figure 10) compared to 1–3%. This can be explained by the known antioxidant properties of alginate and the possibility of a synergistic effect with lysozyme [31,32,33], enhancing the antioxidant potential. This effect was also observed in alginate 2% and chitosan 1% beads, where the reduction was 17%, showing that this encapsulation process increased lysozyme activity. An increase in the antioxidant activity (30%) was observed with lysozyme in the calcium alginate 2% films, while in the calcium alginate 2%-chitosan 1% films was about 20%.

The integration of chitosan, lysozyme, and nano-silver as antimicrobial agents in edible protective hydrosols applied to meat surfaces demonstrated that lysozyme enhances antioxidant properties, while the molecular weight of chitosan significantly influences antioxidant activity [34,35,36]. The most effective formulations were hydrosols containing 1% lysozyme and 2% chitosan, which achieved DPPH radical neutralization rates of 75% and 35%, respectively.

## 3. Conclusions

Lysozyme was successfully encapsulated in beads and films, of calcium alginate 2%, and calcium alginate 2%-chitosan 1%, enabling its gradual release into buffer and water. The encapsulation process, using either calcium alginate alone or a combination of calcium alginate and chitosan, significantly enhanced its antioxidant activity. Additionally, lysozyme in alginate-chitosan beads demonstrated promising anti-inflammatory properties, suggesting potential for future nanoscale delivery systems. However, a key challenge with these systems was the stability of lysozyme. As a protein, lysozyme is susceptible to degradation or denaturation, which may reduce its antibacterial effectiveness, this was not observed in those systems.

## 4. Materials and Methods

### 4.1. Materials and Enzyme

The reagents used in this study were: sodium alginate (Sigma Aldrich, Saint Louis, MO, USA); chitosan (CS, low molecular weight, Sigma Aldrich, Saint Louis, MO, USA); lactic acid (85–90%, VWR, Darmstadt, Germany), lysozyme obtained from hen’s egg white (Lys, ~70,000 U/mg, Sigma Aldrich, Saint Louis, MO, USA), phosphate buffer [potassium dihydrogen phosphate (KH_2_PO_4_, Merk, Darmstadt, Germany) and dipotassium hydrogen phosphate (K_2_HPO_4_, VWR, Darmstadt, Germany)].

Lyophilized *Micrococcus lysodeikticus* (*M. lysodeikticus*, ATCC No. 4698, Sigma-Aldrich, Saint Louis, MO, USA) was used.

All aqueous solutions were prepared with Milli-Q water.

### 4.2. Production of Beads

#### 4.2.1. Calcium Alginate 2%

The following protocol was employed to synthesize beads using a method based on ion exchange and affinity interactions. Initially, a lysozyme-sodium alginate (2%) solution was prepared at a concentration of 0.5 mg/mL, ensuring complete dissolution of the enzyme in the polymer through continuous stirring with a magnetic stirrer. Separately, a 2% calcium chloride solution was prepared using distilled water. The lysozyme-sodium alginate solution was then loaded into a syringe pump, and droplets were carefully dispensed to facilitate bead formation. After 45 min, the beads were collected, and the residual CaCl_2_ solution was analyzed to determine encapsulation efficiency.

#### 4.2.2. Calcium Alginate 2%-Chitosan 1%

**Figure 11:**
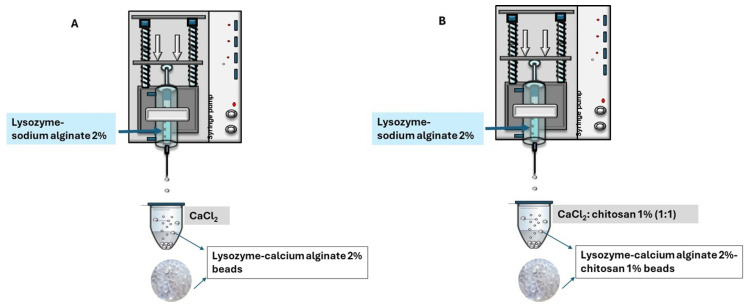
Production of (**A**) lysozyme-calcium alginate 2% beads and (**B**) lysozyme-calcium alginate 2%-chitosan 1% beads.

A lysozyme-sodium alginate (2%) solution was prepared at a concentration of 0.5 mg/mL, ensuring complete dissolution of the enzyme. A 1% chitosan solution was obtained by dissolving medium molecular weight chitosan in 1% lactic acid at 80 °C under constant stirring. This chitosan solution was mixed with a previously prepared 2% calcium chloride solution in a 1:1 ratio. The resulting CaCl_2_-chitosan mixture was maintained at 350 rpm on a magnetic stirrer. Simultaneously, the lysozyme-alginate solution was loaded into a syringe connected to a precision syringe pump and dispensed dropwise into the stirring CaCl_2_-chitosan mixture to promote the formation of uniform hydrogel beads (Figure 11). After 45 min, the beads were collected, and the residual CaCl_2_ solution was analyzed to assess encapsulation efficiency.

### 4.3. Production of Films

#### 4.3.1. Calcium Alginate 2%

**Figure 12:**
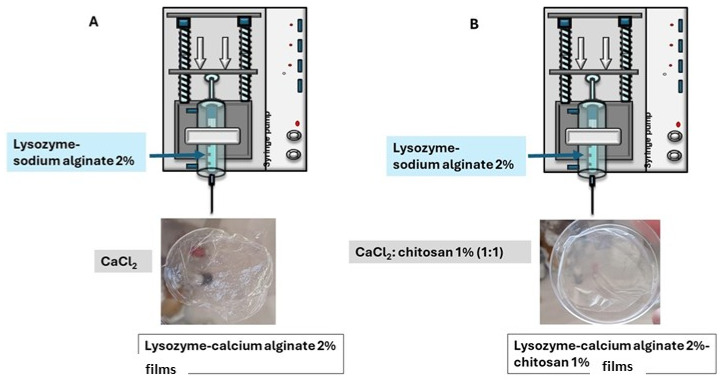
(**A**) Calcium alginate 2% films loaded with lysozyme and (**B**) calcium alginate 2%-chitosan 1% films loaded with lysozyme.

A 0.5 mg/mL lysozyme solution was prepared in 2% (*w*/*v*) sodium alginate. The concentrations of calcium alginate (2%) were settled by weighing 2 g of sodium alginate and solubilized in 100 mL of water. After, the solution was dropped to 100 mL of CaCl_2_ (2%) and the beads were of calcium alginate 2% (*w*/*v*). A 1 mL aliquot of this solution was transferred to a Petri dish, homogenized, and uniformly spread across the surface. Subsequently, 4 mL of a 2% (*w*/*v*) calcium chloride solution was added and allowed to react at room temperature for 30 min to promote crosslinking. Following this incubation period, the calcium chloride solution was removed, and the resulting film was left to dry at room temperature overnight (Figure 12).

#### 4.3.2. Calcium Alginate 2%-Chitosan 1%

A 1% (*w*/*v*) chitosan solution was prepared by dissolving 1 g of medium molecular weight chitosan in 100 mL of lactic acid 1% (*v*/*v*) at 80 °C under continuous stirring. This solution was mixed in a 1:1 ratio with a previously prepared 2% (*w*/*v*) calcium chloride solution.

Separately, a 0.5 mg/mL lysozyme solution was prepared in 2% (*w*/*v*) sodium alginate. A 1 mL aliquot of this solution was transferred to a Petri dish, homogenized, and evenly spread. Subsequently, 4 mL of the chitosan-calcium chloride solution was added and allowed to react at room temperature for 30 min. After the incubation period, the chitosan-calcium chloride solution was removed, and the formed film was left to air dry overnight at room temperature (Figure 12).

### 4.4. Characterization of the Beads and the Films

The morphology of the beads and the films was evaluated by electron microscopy (SEM). For SEM observation, 0.5 cm × 0.5 cm squares of films obtained through the different procedures were cut randomly and analyzed. The beads and films were further characterized using a scanning electron microscopy-field emission gun (FEG-SEM) with a JEOL microscope, model JSM-7001F, operating at 10.0 kV. The surfaces were previously sputter-coated with a gold layer 20 nm thick to avoid charging effects during observation.

### 4.5. Encapsulation Performance 

To confirm the successful encapsulation of lysozyme, it was essential to verify if the enzyme was present in the CaCl_2_ solution. This was assessed using the Bradford assay, following the previously established protocol: 400 µL of Bradford reagent was mixed with 40 µL of the CaCl_2_ solution, and the absorbance was measured at 595 nm, at least in triplicate. A blank sample was included for comparison to ensure accurate measurements.

### 4.6. Lysozyme Activity Assays

The activity of lysozyme was evaluated based on its ability to degrade *Micrococcus lysodeikticus* (Sigma-Aldrich). Standard lysozyme solutions were prepared in distilled water at concentrations of 0.5 mg/mL, 0.25 mg/mL, 0.125 mg/mL, 0.1 mg/mL, 0.0625 mg/mL, and 0.03125 mg/mL.

To assess the effect of *M. lysodeikticus* concentration on the assay, 750 µL of bacterial suspension at different concentrations (3 mg/mL, 2.5 mg/mL, 2 mg/mL, 1.5 mg/mL, 1 mg/mL, and 0.5 mg/mL) was mixed with 250 µL of each lysozyme solution. Lysozyme activity was evaluated at room temperature by monitoring the absorbance decrease at 450 nm every minute for 15 min following the addition of the *M. lysodeikticus* suspension. Absorbance measurements were recorded using a spectrophotometer, and reaction slopes were calculated for each sample. A calibration curve was made by plotting the obtained slopes against lysozyme concentration.

### 4.7. Release Assays

#### 4.7.1. Beads

To evaluate the release kinetics of lysozyme from the hydrogel beads and its retention of antimicrobial activity, 2 g of beads were suspended in 2.5 mL of *Micrococcus lysodeikticus* suspension (1 mg/mL) within a glass container covered with aluminum foil. The container was incubated at 37 °C under agitation at 150 rpm for 24 h. At predefined time points, 1 mL of the sample solution was withdrawn, its absorbance measured at 450 nm, and subsequently returned to the container to maintain a constant volume. Absorbance measurements were performed every 15 min during the first two hours, every 30 min during the third hour, and hourly up to the sixth hour, with additional measurements at 23 and 24 h. The experiments were conducted in triplicate.

To assess lysozyme release under physiological conditions, beads were incubated in water (pH 7.02) and phosphate buffer (pH 7.36) under identical conditions (37 °C, 150 rpm, 24 h). Protein release was monitored by measuring absorbance at 260 nm and 280 nm. For each test medium, 2 g of beads were immersed in 2.5 mL of solution, and 1 mL aliquots were withdrawn at predefined intervals, analyzed spectrophotometrically, and reintroduced into the container to maintain the total volume. Sampling intervals followed the same schedule as described above.

#### 4.7.2. Films

A film was placed in a Petri dish and immersed in 2.5 mL of a Micrococcus lysodeikticus suspension (1 mg/mL). The sample was incubated under the same conditions as the bead experiments (37 °C, 150 rpm, 24 h), and absorbance at 450 nm was measured at predefined time points. After each measurement, the sample was returned to the container to maintain a constant volume. This assay was conducted in triplicate.

The same procedure was applied to assess lysozyme release under physiological conditions. A film was placed in a Petri dish and immersed in 2.5 mL of each test solution (water, pH 7.0; phosphate buffer, pH 7.4). Absorbance was measured at 260 nm and 280 nm at the same time intervals as in the bead experiments. These assays were also performed in triplicate.

### 4.8. Analytical Methods

The protein content in the medium was determined by employing the Bradford method, using a microscale approach adapted to a micromethod [37] for faster multiple sample processing. In this adapted method, 50 µL of Bradford reagent (Coomassie Brilliant Blue dye) was added to 100 µL of the sample to be tested in a microplate (Thermo Scientific Nunc^TM^ 96-well microplates, Thermo Scientific, Essex, UK). The reaction developed for 5 min, and absorbance was read at 595 nm in a microplate reader (BMG LABTACH Fluostar Omega, Biogen Cientifica S.L., Madrid, Spain). The calibration curve for protein quantification was obtained using BSA standard solutions.

UV absorbance at 260/280 nm was the other method used to quantify proteins. It is widely used for the identification of purified proteins with amino acids with aromatic side chains (e.g., tryptophan, tyrosine), it is a fast method, easy to repeat, not consume the protein, and does not require any additional reagents, standards, or incubations [38].

Different lysozyme standard solutions (1.5 mg/mL, 1.0 mg/mL, 0.75 mg/mL, 0.50 mg/mL, 0.25 mg/mL, 0.15 mg/mL 0.125 mg/mL,0.1 mg/mL, 0.0625 mg/mL, 0.03125 mg/mL) were prepared in distilled water and 1 mL of each solution was put into cuvettes and was measured in BioPhotometer (Eppendorf, Hamburg, Germany).

#### 4.8.1. Anti-Inflammatory Evaluation

The anti-inflammatory activity of a sample was determined based on its capacity to inhibit albumin thermal denaturation as described by Mizushima and Kobayashi [39]. The chosen method was an adaptation of the one illustrated by Chandra et al. [40].

A 1% BSA solution (25 mL) in distilled water was prepared. For sample analysis, 1 mL of the BSA solution was added to 200 µL of the sample in an Eppendorf tube. This mixture was then incubated at 37 °C for 15 min followed by a water bath at 70 °C for 5 min. Additionally, a blank was prepared with distilled water instead of the sample extract. Finally, absorbances were read at 660 nm against the blank. The control assay was conducted accordingly with 1 mL of the albumin solution, and the absorbance was read against distilled water. Subsequently, the anti-inflammatory activity was assessed by the inhibition percentage of protein denaturation calculated through Equation (1).

(1) $$Inhibition\;\left(\%\right)=\left(\frac{A_{1}-A_{0}}{A_{0}}\right)\times 100$$

Equation (1)—Inhibition of protein denaturation, where *A*_0_ is the control assay absorbance and *A*_1_ is the sample absorbance [41].

#### 4.8.2. Evaluation of Antioxidant Activity

The antioxidant activity was determined by employing the DPPH method [29]. The DPPH method is based on the color change in the radical species 2,2-difenil-1-picrilhidrazil (DPPH) from purple to pale yellow when reduced. This reaction’s velocity is directly proportional to the antioxidant activity of the compounds present. The absorbance was measured at 517 nm [42,43]. To evaluate the antioxidant activity, 100 µL of the sample was added to 1.4 mL of 100 µM DPPH stock solution in an Eppendorf tube (2 mL). The absorbances at 517 nm were then read at times 0, 10, 20, and 30 min against the blank (ethanol). The samples were protected from the light while the reaction took place. Subsequently, the antioxidant activity was assessed by the scavenging percentage of the DPPH radical calculated according to Equation (2):

(2) $$DPPH\;radical\;scavenging\;activity\;(\%)=\left(\frac{A_{0}-A_{t}}{A_{0}}\right)\times 100$$

Equation (2)—DPPH radical scavenging activity, where *A*_0_ is the time 0 min absorbance and *A_t_* is the absorbance at a given time, *t* min, adapted from [44].

### 4.9. Statistical Analysis

All data were repeated at least 3 times. The representativeness of the data was presented by the mean ± standard deviation.

## Data Availability

The datasets presented in this article are readily available.
